# Distinct Symptoms and Underlying Comorbidities with Latitude and Longitude in COVID-19: A Systematic Review and Meta-Analysis

**DOI:** 10.1155/2022/6163735

**Published:** 2022-01-27

**Authors:** Yong Tian, Qian Wu, Hongwei Li, Qi Wu, Yi Xie, Li Li, Huaiyong Chen

**Affiliations:** ^1^Department of Rehabilitation Medicine, Haihe Hospital, Tianjin University, Tianjin, China; ^2^Department of Respiratory Medicine, Haihe Hospital, Tianjin University, Tianjin, China; ^3^Department of Prevention, Haihe Clinical School, Tianjin Medical University, Tianjin, China; ^4^Key Research Laboratory for Infectious Disease Prevention for State Administration of Traditional Chinese Medicine, Tianjin Institute of Respiratory Diseases, Tianjin, China; ^5^Department of Basic Medicine, Haihe Hospital, Tianjin University, Tianjin, China; ^6^Department of Basic Medicine, Haihe Clinical School, Tianjin Medical University, Tianjin, China; ^7^Tianjin Key Laboratory of Lung Regenerative Medicine, Tianjin, China

## Abstract

The coronavirus disease 2019 (COVID-19) pandemic is straining global health resources, and the prevalence of severe disease appears to vary across countries. In accordance with PRISMA guidelines, we performed a systematic review and meta-analysis of clinical features and underlying medical conditions of COVID-19. Eighty-seven studies, involving 1,434,931 COVID-19 patients from the Americas, Asia, Europe, and Oceania, were included. Geographically, the rate of severity was highest in Asia (95% confidence interval (CI) 0.23‒0.30). The rates of comorbidities of COVID-19 patients in the Americas were significantly higher than those in Asia. Most Asian patients had fever (95%CI 0.70‒0.81), and most Oceanian patients had cough (95%CI 0.68‒0.70) as their prevalent symptom. Dyspnea was common in the Americas (95%CI 0.33‒0.64), Europe (95%CI 0.29‒0.64), and high latitude regions (95%CI 0.53‒0.82). European patients exhibited significantly high rates of loss of smell and taste (95%CI 0.60–0.97). In low-latitude regions, cancer (95%CI 14.50‒4.89) had the strongest correlation with illness severity. Comorbid diseases and clinical manifestations of severe COVID-19 patients vary substantially between latitudes and longitudes. Region-specific care should be considered to treat and improve the prognosis of COVID-19 patients.

## 1. Introduction

Coronavirus disease 2019 (COVID-19) is caused by severe acute respiratory syndrome coronavirus 2 (SARS-CoV-2) [1]. Because of human-to-human transmission, SARS-CoV-2 has become a global pandemic. As of April 28, 2021, the virus has infected 149 million patients globally, causing over 3 million deaths.

Although clinically targeted therapy includes antiviral drugs, convalescent plasma, and monoclonal antibodies, their efficacy and safety remain controversial after assessment in clinical trials [2, 3]. As a nontargeted intervention, traditional Chinese medicine was prescribed to boost immunity against SARS-CoV-2 in China [4, 5]. Mesenchymal stem cell (MSC) therapy was proposed to reduce acute lung injury and was tested in clinical trials to balance the inflammatory response induced by SARS-CoV-2, especially in severe COVID-19 patients [6, 7]. More often, supportive and intensive cardiopulmonary assistance is used to minimize symptomatic progression of the illness.

Symptoms associated with SARS-CoV-2 infection vary substantially from person to person. While some patients remain asymptomatic, the main clinical features of symptomatic COVID-19 patients include fever, fatigue, muscle ache, cough, sore throat, nausea, abdominal pain, anorexia, loss of olfactory taste, vomiting, diarrhea, rhinitis, and dyspnea [8–10]. Over 20% of COVID-19 patients develop acute respiratory distress syndrome, leading to respiratory failure or even death [11]. There are many risk factors for severe morbidity and mortality in COVID-19 patients, such as sex, age, and underlying medical conditions, including coronary heart disease, diabetes, hypertension, lung diseases, and cancer [12, 13].

Previous related systematic reviews and meta-analyses have been performed to assess clinical features, underlying medical conditions, laboratory and medical imaging findings, illness severity, and fatal outcomes, in individual countries [14–16]. However, genetic background, environmental factors, diagnostic criteria, clinical management practices, and treatment choices may also contribute to disease manifestations and progression in different regions.

In this study, we analyzed the published scientific literature on COVID-19 patients in geographical regions, including the Americas, Asia, Europe, and Oceania, concerning the clinical characteristics of COVID-19 as well as its severity and risk of underlying medical conditions. We hypothesized that region-specific symptoms and population-specific comorbidities may be discovered to optimize treatment options and to improve care for COVID-19 patients, in a situation where no curable treatments are currently available.

## 2. Methods

### 2.1. Search Strategy and Selection Criteria

To perform a meta-analysis, we performed a literature search of articles in Embase and PubMed, published between Jan 1, 2020, and Jun 30, 2020, without language restrictions. We used the search terms “COVID-19,” “SARS coronavirus 2,” and “coronavirus disease 2019.” Reports were limited to human studies. The full search strategy is shown in Table S1.

To be eligible for inclusion, the study subjects were laboratory-confirmed patients with COVID-19, and the patients' symptoms or underlying medical conditions had to be reported. As our study focused on comparing the clinical symptoms and underlying comorbidities of COVID-19 patients in different areas, we excluded studies that only reported on specific populations, such as patients in the ICU, children, or medical staff, and excluded case reports to avoid the associated bias. Multiple studies that reported on the same group of participants were identified based on the admission hospital and period of hospitalization of participants, and the report that best provided our observational indicators was included for further analysis. All included studies had to be published online or in print as full reports. The eligibility of the studies was independently assessed by two investigators (YT and YX).

Studies were screened and managed using EndNote X 9.0 software. We used Microsoft Excel spreadsheet for recording extracted data. We used the Newcastle‒Ottawa Scale to evaluate the quality of the studies: a score higher than 5 was considered high-quality, and low-quality studies were excluded.

The study process was in accordance with the operating procedures in the PRISMA guidelines. The protocol for this meta-analysis was established before the analyses and was registered in PROSPERO (CRD42020203520).

### 2.2. Data Analysis

We extracted the associations of the severity of COVID-19 with sex, age, comorbidities, and clinical symptoms and performed subgroup analyses and meta-regression analyses by geographic location (longitudes and latitude) to explore heterogeneity between studies. Patients labeled “Severe” or “ICU” in the included studies were considered as severe patients (specific strategies are included in the Appendix). Because research data from Africa was limited, only studies on COVID-19 patients from both Americas, Asia, Europe, and Oceania were analyzed. The latitude was divided into low latitude (north/south latitude 0° to north/south latitude 30°), middle latitude (30° north/south latitude to 60° north/south latitude), and high latitude (60° north/south latitude to 90° north/south latitude).

All analyses were conducted using STATA 15 (http://www.stata.com). The command “Metaprop” was used to calculate the prevalence and proportions (95% CI) of the single arm meta-analysis. Random effects of meta-analysis was performed to obtain summary effect measures as high between-study heterogeneity was expected. Between-study heterogeneity was evaluated using the *I*^2^ statistic with a *p* value < 0.10 to define significant heterogeneity [17]. Publication bias of the included studies was assessed with Egger's test [18]. We also performed sensitivity analysis to assess the stability of the results. The effects of each study were investigated by the leave-one-out approach, and no signs of bias were found.

### 2.3. Role of the Funding Source

This work was supported by the Science and Technology Planning Project of Tianjin Jinnan District (20200117). This work was also supported by the Science and Technology Projects in Key Fields of the Department of Traditional Chinese Medicine, Tianjin Municipal Health Commission (Project Number: 2021011). Corresponding authors had access to all data.

## 3. Results

**Figure 1:**
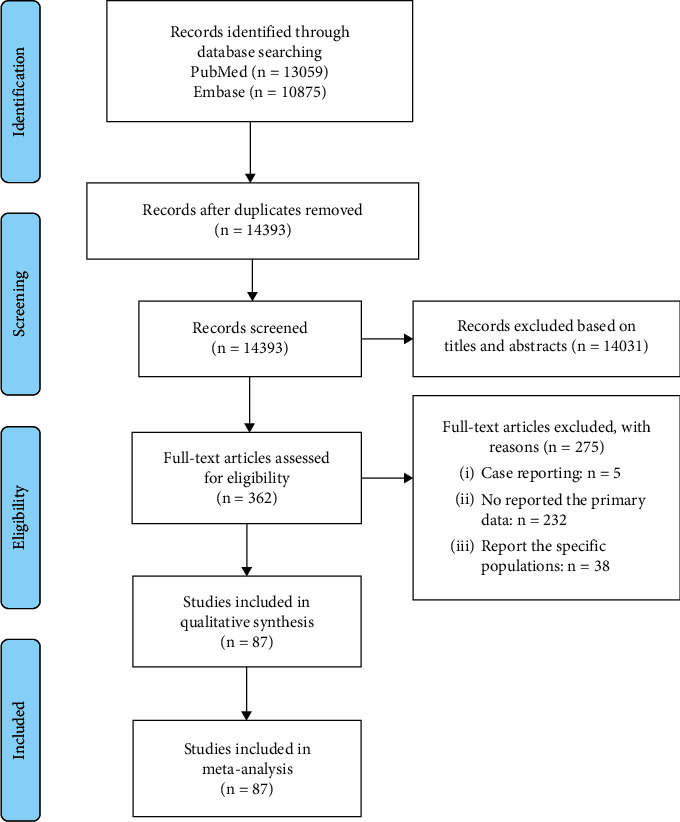
Study selection process.

The search strategy generated 23,934 citations, but 14,393 articles were left after duplicates were removed (Figure 1). Of these, 14,031 studies were excluded after a review of the title and abstract. After reading the full text of the remaining 362 studies, we found that 87 studies, representing 1,349,931 COVID-19 patients, fulfilled the eligibility criteria. Among these, 12 studies were from the Americas (United States: 9, Canada: 1, Bolivia: 1, Mexico: 1), 61 studies from Asia (China: 51, South Korea: 4, Japan: 2, Singapore: 1, Iran: 1, India: 1, Iraq: 1), 12 studies from Europe (Italy: 5, France: 2, Norway: 1, Switzerland: 1, the United Kingdom: 2, multiple European countries merged: 1), and 2 studies from Oceania (Australia: 2) (Tables S2, and S3).

### 3.1. Proportion of Severe COVID-19 Patients

**Figure 2:**
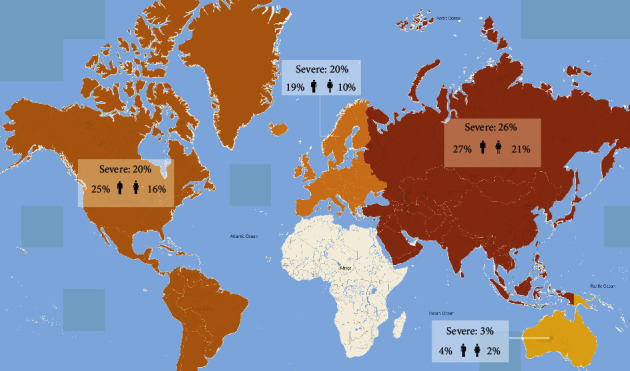
The severity rate of COVID-19 patients in different regions.

Of the 87 studies, 51 reported the severity rate of COVID-19 patients. The rate ranged from 26% in Asia, 20% in the Americas and Europe, and 3% in Oceania, with a pooled rate of 24% (95%CI 20‒30, *I*^2^ = 99.8%) (Figure 2, Table S4). The severity rate in Oceania was the lowest among all region studied (20%, 95%CI 12‒29). Twenty-six percent of male COVID-19 patients (95%CI 20‒30%) developed severe symptoms, compared to 19% of female patients (95%CI 14‒24%); this gender trend was seen in COVID-19 patients from all four regions (Figure 2, Table S4). SARS-CoV-2 infection in the low-latitude regions showed a tendency for a high prevalence of severe cases among both men and women (Figure 2, Table S4).

**Figure 3:**
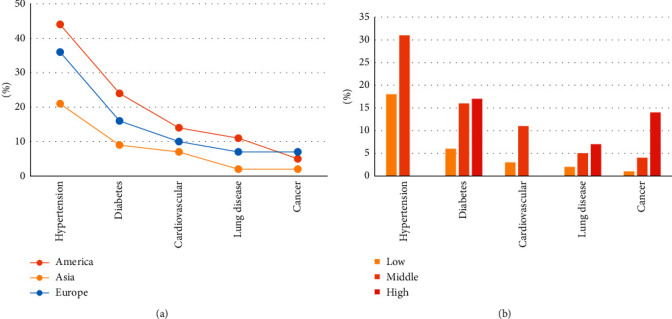
Comorbidities of COVID-19 patients in different regions.

Sixty studies, comprising 1,418,194 COVID-19 patients, reported comorbidities. Hypertension was the most common comorbidity in the pooled COVID-19 patients, accounting for 26% (95%CI 22‒31%), followed by diabetes (13%, 95%CI 10‒15%), cardiovascular diseases without hypertension (8%, 95%CI 7‒10%), lung diseases (4%, 95% CI 4‒5%), and cancer (3%, 95%CI 2‒4%) (Figure 3). Geographically, the rate of comorbidities among COVID-19 patients in the Americas was significantly higher than that among patients in Asia, and the prevalence of cancer and lung diseases among COVID-19 patients in Europe was significantly higher than that among patients in Asia (Figure 3, Table S5). The proportion of comorbidities in COVID-19 patients showed an upward trend with the increase in latitude (Figure 3).

In the overall comparison of mild and severe cases, diabetes (odds ratio [OR] 2.70, 95%CI 1.96‒3.71) and cardiovascular diseases (OR 2.62, 95%CI 1.22‒5.64) were more closely related to severe cases. Hypertension (OR 2.08, 95%CI 1.26‒3.42) and cancer (OR 2.07, 95%CI 1.75‒2.57) were more closely related to severe cases than lung diseases (OR 1.79, 95%CI 1.15‒2.79) (Table S6).

In Asia, hypertension (OR 2.77, 95%CI 1.27‒4.07), cardiovascular disease (OR 2.91, 95%CI 2.14‒3.94), lung disease (OR 2.11, 95%CI 1.15‒3.88), and cancer (OR 2.68, 95%CI 2.15‒3.34) were more clearly correlated with the severity of COVID-19 than in other regions (Table S7). In contrast, diabetes (OR 3.41, 95%CI 1.71‒6.78) showed the strongest correlation with the severity of COVID-19 in the Americas. There was no significant correlation between comorbidities and COVID-19 severity among Europeans (Table S7). In latitude-level comparisons, the low-latitude countries showed the strongest correlation between cancer and severe COVID-19 (OR 4.72, 95%CI 14.50‒4.89), and the rate of each comorbidity among COVID-19 patients was higher than that in the mid-latitude countries (Table S7).

### 3.2. Prevalence of Severity and Clinical Manifestations

**Figure 4:**
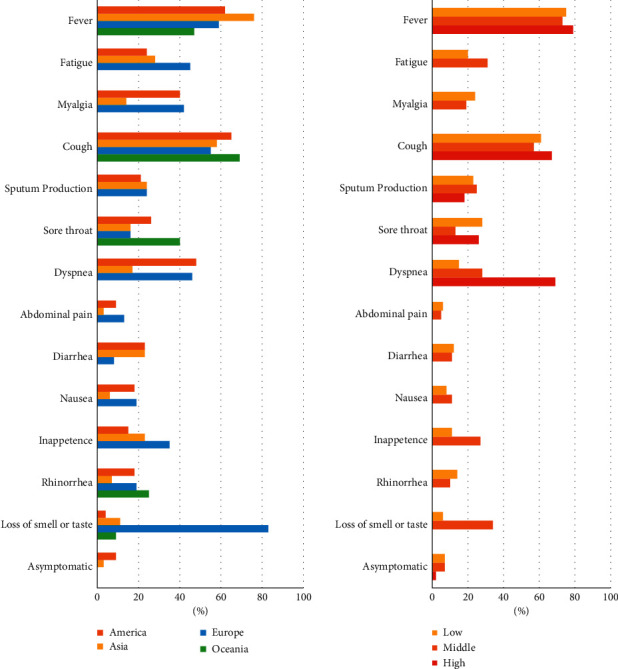
Clinical manifestations of COVID-19 patients in different regions.

Fever (75%, 95%CI 67‒75%) and cough (58%, 95%CI 55‒62%) were the most common clinical manifestations in all COVID-19 patients, with the highest rate of fever in Asia (76%, 95%CI 70‒81%) and the highest rate of cough (69%, 95%CI 68‒70%) in Oceania (Figure 4). Dyspnea was more common in COVID-19 patients from the Americas (48%, 95%CI 33‒64%), Europe (49%, 95%CI 29‒64%), and high-latitude countries (69%, 95%CI 53‒82). In addition, European COVID-19 patients experienced significantly higher rates of loss of olfaction and taste (83%, 95%CI 60‒97%) and upper digestive tract symptoms, such as nausea (19%, 95%CI 17‒21%) and loss of appetite (35%, 95%CI 28‒42) than in other regions (Figure 4, Table S5).

**Figure 5:**
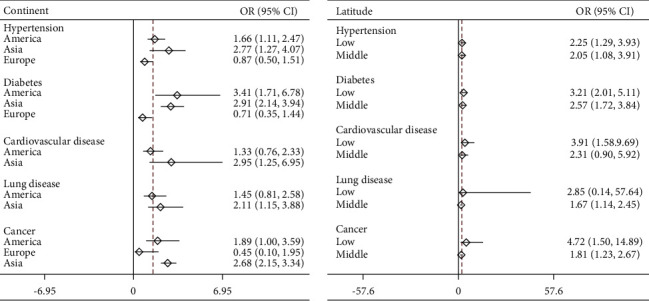
Comparison of the comorbidities of mild and severe COVID-19 patients in different areas. Hypertension includes primary hypertension and secondary hypertension; diabetes includes type 1 diabetes and type 2 diabetes; cardiovascular disease includes coronary artery disease, arrhythmia, congestive heart failure; lung disease includes COPD, asthma, obstructive sleep apnea; cancer includes all kinds of cancer.

In the overall comparison of common and severe COVID-19 patients, dyspnea (OR 6.49, 95%CI 3.60‒11.72), abdominal pain (OR 2.22, 95%CI 1.17‒4.23), and fatigue (OR 1.83, 95%CI 1.48‒2.27) correlated strongly with illness severity (Table S7). Cough (OR 1.12, 95%CI 0.78‒1.62), diarrhea (OR 1.19, 95%CI 0.79‒1.78), and myalgia (OR 1.25, 95%CI 0.98‒1.60) were not significantly related to the severity of COVID-19 (Figure 5).

**Figure 6:**
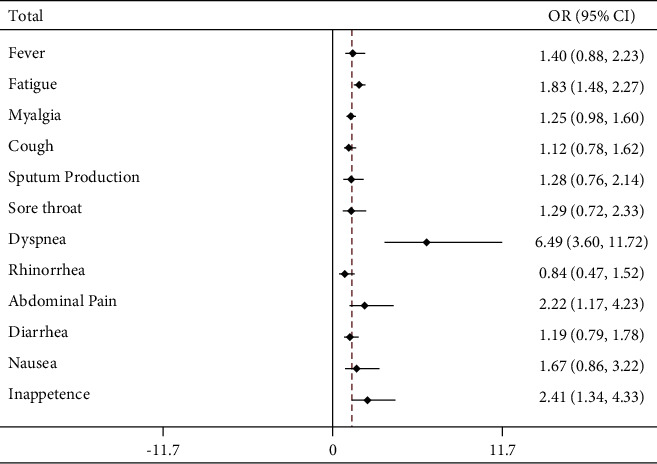
Clinical manifestations of mild and severe COVID-19 patients.

In Asia, dyspnea (OR 9.55, 95%CI 4.67‒19.54), fatigue (OR 1.83, 95%CI 1.48‒2.27), and anorexia (OR 2.41, 95%CI 1.34‒4.33) showed some association with COVID-19 severity (Table S7). Dyspnea (OR 2.0, 95%CI 1.29‒3.08) and abdominal pain (OR 3.61, 95%CI 1.21‒10.72) were related to the severity of COVID-19 in the Americas (Figure 6, Table S7).

**Figure 7:**
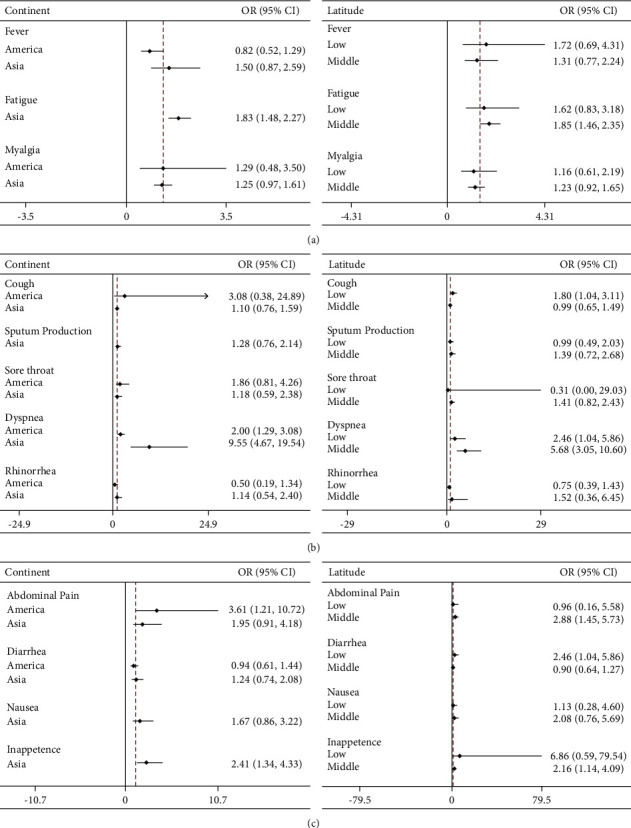
Comparison of the clinical manifestations of mild and severe COVID-19 patients in different regions.

In COVID-19 patients from low-latitude countries, dyspnea (OR 2.46, 95%CI 1.04‒5.86) and diarrhea (OR 2.46, 95%CI 1.04‒5.86) were closely related to the disease severity, but in mid-latitude regions, patients manifested mainly with fatigue (OR 1.85, 95%CI 1.46‒2.35), dyspnea (OR 5.68, 95%CI 3.05‒10.61), abdominal pain (OR 2.88, 95%CI 1.45‒5.73), and anorexia (OR 2.16, 95%CI 1.14‒4.09) (Figure 7, Table S7).

## 4. Discussion

In current systematic review and meta-analysis, we included clinical symptoms, underlying comorbidities, and the severity of COVID-19 in patients from the Americas, Asia, Europe, and Oceania, reported in 87 studies. COVID-19 data for Africa were sparse in the first half of 2020 and thus were not included in this review. We found that Asian COVID-19 patients had the highest proportion of fever, Oceania patients had the highest proportion of cough, sore throat, and rhinitis, and European patients more frequently had smell and taste loss. Patients from the Americas patients had the highest proportion of comorbidities, such as cardiovascular disease, hypertension, and diabetes than those from other regions. The proportion of severely ill patients was the highest in Asia, but the lowest in Oceania. This analysis provided a reference for the rapid and accurate identification of COVID-19 patients in different geographical locations and can facilitate timely treatment, and reduce the occurrence of severe cases.

The proportion of comorbidities, such as cardiovascular and diabetes, in COVID-19 patients from America was significantly higher than that of patients from other regions. Cardiovascular disease and diabetes are closely related to lifestyle and dietary habits. Obesity is considered to be the main risk factor affecting the morbidity and mortality associated with metabolic diseases, such as cardiovascular and diabetes [19]. American countries, including the United States, Mexico, and Canada, have a high obesity rate [20]. In 2015, the average prevalence of obesity among adults was 38.2% in the United States, much higher than global average (19.5%) based on the Obesity Update 2017 (http://www.oecd.org/health/obesity-update.html). A high carbohydrate intake is reported to be the dietary factor most related to the risk of cardiovascular disorders and death [21, 22]. Both the obesity rate and carbohydrate intake in the Americas are significantly higher than those in Asia, which may explain the higher rate of comorbid hypertension, cardiovascular disease, and diabetes in COVID-19 patients in the Americas than in those in Asia.

COVID-19 patients from the Americas predominantly seemed to suffer from abdominal pain and diarrhea. Some studies have reported an imbalance of intestinal flora diversity in COVID-19 patients, where the level of probiotics was reduced [23], but the level of opportunistic pathogens was increased [24]. SARS-CoV-2 nuclei acid has been detected in the stool of COVID-19 patients [25]. Intestinal ACE2 is a chaperone of the amino acid transporter B0AT1 and plays an essential role in the transmission of intestinal epithelial amino acids. The B0AT1/ACE2 complex regulates the intestinal microbiota [26], and dysregulation of this complex may thus cause imbalanced flora in COVID-19 patients, resulting in diarrhea and other intestinal symptoms [27]. In addition to high carbohydrate levels, the typical American diet is high in fat and protein and *Firmicutes* species predominate in the intestines of individuals living in the USA. However, the typical Asian diet is rich in fiber, and *Bacteroides* and *Actinomycetes* species are more common in the intestines of Asian populations [28, 29]. The diversity of the diet influences the gut microbiota, which may indirectly affect the digestive tract reaction to SARS-CoV-2 [30].

The present study found that the incidence of fever in COVID-19 patients was highest in Asia. This could be due to diagnostic criteria for fever, which vary from region to region. The temperature threshold that defines fever symptoms is lowest in Asia. In China, an oral temperature of 37.3°C was set as the threshold temperature for fever in the diagnosis of COVID-19. In Japan, Singapore, and South Korea, fever was defined as a body temperature of 37.5°C or higher [31, 32]. In the USA, the American Academy of Infectious Diseases defined fever when the oral temperature reached 38.3°C [33]. Europe considered fever when the oral temperature exceeded 37.8°C [34]. In addition, fever was the most common symptom at the beginning of the pandemic. In China, fever clinics have been established across the country. All patients who visited the fever clinic were requested to undergo nucleic acid testing for SARS-CoV-2. COVID-19 patients with fever could be identified and hospitalized in the most timeous manner.

COVID-19 patients in Australia seemed to have a significantly higher proportion of sore throat, cough, and rhinitis. Environmental factors, such as pollen, damp buildings, and mold exposure, have many potential impacts on human health and affect the occurrence of respiratory diseases in particular [35, 36]. These environmental factors lead to allergies and immune responses, increasing the respiratory disease burden [37]. Pollen and fungal spores are particularly prominent in Australia, and the incidence of bronchial asthma in Australia is 21.0%, much higher than the global average of 4.3% [38]. Upper respiratory tract symptoms may be related to the environmentally driven hyperresponsiveness in the upper airways of COVID-19 patients in Australia.

Taste and olfactory disorders were more prominent in Europe COVID-19 patients than in other regions. SARS-CoV-2 has potential nerve invasion ability [39]. SARS-CoV-2, by binding to ACE2, causes dysfunction of the olfactory bulb and damage to the epithelial cells of the mucosa of the oral cavity [40, 41]. In addition, the gene *ACE2* contains multiple single nucleotide variants, which affects its expression in various tissues, directs a different response to SARS-CoV-2 infection, and leads to development of a distinct COVID-19-related phenotype [42]. The distribution of variants differ in frequencies in different brain tissues, indicating a possible link between *ACE2* genetic variability and taste and olfactory disorders in COVID-19 patients [42]. The frequency of the rs2285666 mutation is lower in the European population (0.235) than in the Chinese population (0.556) and the American population (0.33) [43]. Future research is needed to clarify whether these *ACE2* variants confer taste and olfactory disorders in the global population or only in the European population.

Our data have implications for both clinical practice and future research. First, the diarrhea symptoms of COVID-19 patients from the Americas and Asia are prominent, which prompts the disease control centers in these regions to monitor and properly dispose of patients' excrement to prevent the secondary spread of the virus. It may be worth investigating whether supplementation of these COVID-19 patients with flora regulators could relieve gastrointestinal symptoms and accelerate recovery. Second, most severe COVID-19 patients are elderly, and thus, clinical management of these patients with underlying comorbidities becomes increasingly important during this pandemic. Third, numerous studies have indicated that ACE2 is expressed in the respiratory tract, digestive tract, and nervous system [44]. However, further research is needed to address whether or not region-typical COVID-19-related symptoms are related to the variable expression of ACE2 across populations. For example, it would be interesting to investigate whether ACE2 is more abundantly expressed in oral and neural cells in the European population and more highly expressed in the upper airway cells in the Australian population. Fourth, phylogenetic analyses of SARS-CoV-2 genes and proteins have revealed numerous mutations and multiple clades [45, 46]. Some mutations are located in the receptor-binding domain of the spike glycoprotein, which determines virus virulence and host susceptibility [47]. Mutations in SARS-CoV-2 strains from America, Asia, and Europe differ [47, 48]. How these mutations impact host infection by SARS-CoV-2 and the clinical symptoms need to be investigated in future.

In summary, our research found that the clinical manifestations and comorbidities of COVID-19 patients in different latitudes and longitudes are quite different. We believe that differences in the population's genetics, population composition, and national control efforts in different latitudes and longitudes may be the reasons for the differences in the clinical manifestations and comorbidities of COVID-19 patients in various regions. To treat COVID-19 patients more effectively, the regional differences of patients should be considered, so that patients can be identified and treated more timely and accurately.

The limitations of this study include the fact that COVID-19 clinical data for Africa were sparse in the time-window of this study (January 1 to June 30, 2020). During this period, clinical data of COVID-19 patients in many countries had not yet been published, and thus the number of enrolled countries from each geographical region was not large. Fewer COVID-19 cases were collected from high-latitude regions than from low-latitude regions. Some included studies failed to present a complete comparison of clinical symptoms. In addition, laboratory indicators that distinguished severe symptoms from moderate symptoms were diverse due to the source of testing reagents. The number of COVID-19 patients included from each region varied markedly, which may have caused bias in the comparisons. In addition, we included only adult COVID-19 patients, excluding special populations, including children and pregnant women, given our research goal. These factors may all have confounded the data representation of each region to various extents.

In summary, this systematic review and meta-analysis showed distinct clinical symptoms and various underlying medical conditions of COVID-19 patients in different regions, which may be related to factors such as the diversity and evolution of SARS-CoV-2, host genetic factors, climate environment, and lifestyle. Identifying the typical clinical characteristics of COVID-19 patients in different regions may facilitate formulation of more targeted prevention and treatment strategies. The pandemic has not yet been effectively controlled. This analysis of the global characteristics of COVID-19 will deepen our understanding of the disease and assist in directing an efficient response to the early treatment of the disease. It is essential to move the early warning window of illness severity forward to reduce the incidence of severe cases, treat severe cases effectively, reduce mortality, and provide clues for the control of the pandemic.

## 5. Disclosure

An earlier version of this manuscript [49] has been presented as preprint in Research Square according to the following link: https://www.researchsquare.com/article/rs-117666/v1.

## Data Availability

Data can be obtained upon request to the corresponding author Huaiyong Chen.

## Supplementary Materials

Supplementary Materials Table S1: search strategies in databases. Table S2: description of including studies. Table S3: the relationship between the severity rate of COVID-19 patients with gender, age and geographical location. Table S4: comorbidities and clinical manifestations of COVID-19 patients in different regions. Table S5: the relationship between the severity rate of COVID-19 patients with gender, age and geographical location. Table S6: compare the comorbidities of mild and severe COVID-19 patients. Table S7: compare the comorbidities and Clinical manifestations of mild and severe COVID-19 patients in different regions.
