# Supplementary material for: Distinct Symptoms and Underlying Comorbidities with Latitude and Longitude in COVID-19: A Systematic Review and Meta-Analysis
Source: Can Respir J. 2022 Jan 27;2022:6163735. doi: 10.1155/2022/6163735 (PMC8793347; doi:10.1155/2022/6163735)
Supplement: Supplementary Materials — Table S1: search strategies in databases. Table S2: description of including studies. Table S3: the relationship between the severity rate of COVID-19 patients with gender, age and geographical location. Table S4: comorbidities and clinical manifestations of COVID-19 patients in different regions. Table S5: the relationship between the severity rate of COVID-19 patients with gender, age and geographical location. Table S6: compare the comorbidities of mild and severe COVID-19 patients. Table S7: compare the comorbidities and Clinical manifestations of mild and severe COVID-19 patients in different regions. [file 6163735.f1.docx]

**Appendix Tables**

| **Table S1.** Search strategies in databases |
| --- |
| **Embase** |
| ('covid 19':ab,ti OR 'sars coronavirus 2':ab,ti OR 'coronavirus disease 2019':ab,ti OR '2019 novel coronavirus':ab,ti) AND [1-1-2020]/sd NOT [30-6-2020]/sd  Study types limited Humans |
| **PubMed** |
| ((covid-19[Title/Abstract]) OR (sars coronavirus 2[Title/Abstract]) OR (coronavirus disease 2019[Title/Abstract]) OR (2019 novel coronavirus[Title/Abstract])) AND ("2020/01/01"[Date - Publication] : "2020/06/30"[Date - Publication])  Limited：Humans |

**Table S2.** Description of including studies.

| **Study** | **Total patients** | **Language** | **Latitude^*^** | **Country** | **Age*** | **Deadline(mm.yy)** | **Quality** |
| --- | --- | --- | --- | --- | --- | --- | --- |
| **America** |  |  |  |  |  |  |  |
| CDC^1^ | 1320488 | English | - | America | 48(33-63) | Jan.22 to May.30 | 6 |
| Shikha Garg^2^ | 180 | English | Middle | America | NA | Mar.1 to Mar.28 | 6 |
| Jessica Ferguson^3^ | 72 | English | Middle | America | 60.4(43.4-70.6) | Mar.13 to Apr.11 | 6 |
| Safiya Richardson^4^ | 5700 | English | Middle | America | 63(52-75) | Mar.1 to Apr.4 | 6 |
| Jeremy A W Gold^5^ | 305 | English | Middle | America | 60(46-69) | Mar.1 to Mar.30 | 6 |
| Youyou Duanmu^6^ | 100 | English | Middle | America | 45(32–65) | Mar.4 to Mar.23 | 9 |
| Laura C Myers^7^ | 377 | English | Middle | America | 61(50-73) | Mar.1 to Mar.31 | 7 |
| Parag Goyal^8^ | 393 | English | Middle | America | 62.2(48.6–73.7) | Mar.3 to Mar.27 | 6 |
| Geehan Suleyman^9^ | 463 | English | Middle | America | 57.5 | Mar.9 to Mar.27 | 6 |
| Molly Lin^10^ | 135 | English | Middle | Canada | 28(23–49) | Jan.20 to Feb.19 | 6 |
| Juan Pablo  Escalera-Antezana^11^ | 12 | English | Low | Bolivia | 39(25.3-43.3) | Mar.2 to Mar.15 | 6 |
| Edgar Ortiz-Brizuela^12^ | 309 | English | Low | Mexico | 43(33-54) | Feb.26 to Apr.11 | 6 |
| **Europe** |  |  |  |  |  |  |  |
| Jerome R Lechien^13^ | 1420 | English | - | - | 39.17±12.09 | Mar.22 to Apr.10 | 6 |
| Andrea Giacomelli^14^ | 59 | English | Middle | Italy | 60(50-74) | NA | 9 |
| Corrado Lodigiani^15^ | 388 | English | Middle | Italy | 66(55-75) | Feb.13 to Mar.10 | 6 |
| Riccardo M Inciardi^16^ | 99 | English | Middle | Italy | 67±12 | Mar.4 to Mar.25 | 6 |
| Marta Colaneri^17^ | 44 | English | Middle | Italy | 67.5(10-94) | Feb.21 to Feb.28 | 7 |
| Fabio Ciceri^18^ | 410 | English | Middle | Italy | 65(56-75) | Feb.25 to Mar.24 | 8 |
| Jean-Christophe Lagier^19^ | 3737 | English | Middle | France | 45.3 | Mar.3 to Mar.31 | 6 |
| Frédéric Lapostolle^20^ | 197 | English | Middle | France | 44(31-54) | Mar.1 to Mar.17 | 7 |
| Håkon Ihle-Hansen^21^ | 42 | English | High | Norway | 72.5(30-95) | Mar.19 to Mar.31 | 5 |
| Marlene M Speth^22^ | 103 | English | Middle | Switzerland | 46.8 | Mar.3 to Apr.17 | 9 |
| K Khalil^23^ | 220 | English | Middle | England | 66.9 | Mar.7 to Apr.7 | 6 |
| Annemarie B Docherty^24^ | 20133 | English | Middle | England | 73(58-82) | Feb.6 to Apr.19 | 6 |
| **Oceania** |  |  |  |  |  |  |  |
| 2019-nCoV National Incident Room Surveillance Team^25^ | 7135 | English |  | Australia | NA | By May.24 | 6 |
| 2019-nCoV National Incident Room Surveillance Team^26^ | 6600 | English |  | Australia | NA | By May.17 | 6 |
| **Asia** |  |  |  |  |  |  |  |
| Peong Gang Park^27^ | 309 | English | Middle | Korea | NA | By Mar.9 | 5 |
| Eu Suk Kim^28^ | 28 | English | Middle | Korea | 40(20-73) | Jan.19 to Feb.17 | 5 |
| Kyung Soo Hong^29^ | 98 | English | Middle | Korea | 55.4±17.1 |  | 7 |
| Wonjun Ji^30^ | 7341 | English | Middle | Korea | 47.05±19.0 | By May.15 | 7 |
| Yuzo Arima^31^ | 19 | English |  | Japan | NA | Jan.29 to Jan.31 | 5 |
| Sakiko Tabata^32^ | 104 | English |  | Japan | 68(47-75) | By Feb.11 | 6 |
| Mohamad Nikpouraghdam^33^ | 2968 | English | Middle | Iran | 56(46-65) | Feb.19 to Apr.15 | 5 |
| Muayad A Merza^34^ | 15 | English | Middle | Iraq | 28.06 | Mar.18 to Apr.7 | 5 |
| Barnaby Edward Young^35^ | 18 | English | Low | Singapore | 47(31-73) | Jan.23 to Feb.3 | 6 |
| Amit Aggarwal^36^ | 32 | English | Low | India | 54.5(46.25-60) | Apr.10 to Apr.30 | 6 |
| WeiJie Guan^37^ | 1099 | English | - | China | 47(35-58) | By Jan.29 | 8 |
| China CDC^38^ | 44672 | Chinses | - | China | NA | By Feb.11 | 8 |
| Adam Bernheim^39^ | 121 | English | - | China | 45(18-80) | Jan.18 to Feb.2 | 7 |
| Xiaolong Qi^40^ | 70 | English | - | China | NA | By Mar.19 | 9 |
| Sijia Tian^41^ | 262 | English | Middle | China | 47.5 | Jan.20 to Feb.10 | 7 |
| Lijun Sun^42^ | 55 | English | Middle | China | 44(34-56) | Jan.20 to Feb.15 | 6 |
| Xiao Yu^43^ | 333 | English | Middle | China | 50(35-63) | By Feb.19 | 6 |
| Chunxia Cao^44^ | 135 | English | Middle | China | NA | Jan.1 to Feb.24 | 6 |
| Yalei Shang^45^ | 307 | English | Middle | China | 46(33-55) | Jan.10 to Mar.3 | 7 |
| Qian Yu^46^ | 421 | English | Middle | China | NA | Jan.10 to Feb.18 | 6 |
| Rui Huang^47^ | 202 | English | Middle | China | 44(33-54) | Feb.1 | 6 |
| KaiCai Liu^48^ | 73 | English | Middle | China | 5-86 | Jan.21 to Feb.3 | 6 |
| Yan Ma^49^ | 47 | English | Middle | China | 34(1-72) | Jan.23 to Mar.10 | 7 |
| Wenjun Du^50^ | 67 | English | Middle | China | NA | Jan.23 to Feb.15 | 6 |
| Liang Su^51^ | 23 | English | Middle | China | NA | Jan.24 to Feb.24 | 7 |
| Xiaoming Li^52^ | 131 | Chinses | Middle | China | NA | 1Feb.28 to Feb.10 | 5 |
| K. Wang^53^ | 114 | English | Middle | China | 53(27-78) | Jan.25 to Feb.9 | 6 |
| Lei Pan^54^ | 204 | English | Middle | China | 52.9 | Jan.18 to Feb.28 | 9 |
| Kui Liu^55^ | 137 | English | Middle | China | 57(20-83) | 1Feb.30 to Jan.24 | 7 |
| Hong Zhang^56^ | 194 | English | Middle | China | 48.3(33-56) | Jan.22 to Feb.28 | 6 |
| Qiurong Ruan^57^ | 150 | English | Middle | China | NA | By Feb.18 | 6 |
| Ling Mao^58^ | 214 | English | Middle | China | 52.7 | Jan.16 to Feb.19 | 6 |
| Gemin Zhang^59^ | 95 | English | Middle | China | 49.0(39-58) | Jan.16 to Feb.25 | 6 |
| Fei Zhou^60^ | 191 | English | Middle | China | 56(46-67) | By Jan.31 | 6 |
| Dawei Wang^61^ | 138 | English | Middle | China | 56(22-92) | Jan.1 to Jan.28 | 7 |
| Chen, Lei^62^ | 29 | Chinses | Middle | China | NA | By Jan | 5 |
| Nanshan Chen^63^ | 99 | English | Middle | China | 55.5(21-82) | Jan.1 to Jan.20 | 6 |
| Chaolin Huang^64^ | 41 | English | Middle | China | 49(41-58) | By Jan.2 | 7 |
| JinJin Zhang^65^ | 140 | English | Middle | China | 57(25-87) | Jan.21 to Feb.16 | 7 |
| Fang Liu^66^ | 140 | English | Middle | China | 65.5(54.3-73) | Jan.18 to Mar.12 | 6 |
| Qiuxiang Yang^67^ | 136 | English | Middle | China | 56(44-64) | Jan.28 to Feb.12 | 6 |
| Chuan Qin^68^ | 452 | English | Middle | China | 58(47-67) | Jan.10 to Feb.12 | 6 |
| Guqin Zhang^69^ | 221 | English | Middle | China | 55.0(39.0-66.5) | Jan.2 to Feb.10 | 6 |
| Yun Feng^70^ | 476 | English | Middle | China | 53(40-64) | Jan.1 to Feb.15 | 6 |
| XinYing Zhao^71^ | 91 | English | Low | China | 46 | Jan.16 to Feb.10 | 6 |
| Sheng Yin^72^ | 33 | English | Low | China | 46(33.5-65) | Jan.9 to Feb.9 | 6 |
| Jiangshan Lian^73^ | 465 | English | Low | China | 45(5-88) | Jan.17 to Jan.31 | 6 |
| Shufa Zheng^74^ | 96 | English | Low | China | 55(44.3-64.8) | Jan.19 to Mar.20 | 6 |
| Qingqing Chen^75^ | 145 | English | Low | China | 47.5 | Jan.1 to Mar.11 | 6 |
| Yu Shi^76^ | 487 | English | Low | China | 46(19) | By Feb.7 | 7 |
| Wenjie Yang^77^ | 149 | English | Low | China | 45.11±13.35 | Jan.17 to Feb.10 | 6 |
| Suxin Wan^78^ | 135 | English | Low | China | 47(36-55) | Jan.23 to Feb.8 | 6 |
| Peng Chen^79^ | 136 | English | Low | China | 47(37-61) | Jan.25 to Feb.20 | 6 |
| Yongli Zheng^80^ | 99 | English | Middle | China | 49.39±18.45 | Jan.16 to Feb.20 | 7 |
| Xi Xu^81^ | 90 | English | Low | China | 50(18-86) | Jan.23 to Feb.4 | 6 |
| Ying Zhu^82^ | 14 | English | Low | China | NA | Jan.31 to Feb.14 | 5 |
| YingTao Zhang^83^ | 1350 | Chinses | Low | China | 44.1±17.9 | By Mar.4 | 6 |
| Yingxia Liu^84^ | 12 | English | Low | China | NA | By Jan.21 | 5 |
| Kai Liu^85^ | 56 | English | Low | China | NA | Jan.15 to Feb.18 | 6 |
| Iek Long Lo^86^ | 10 | English | Low | China | 54(27-64) | Jan.21 to Feb.16 | 6 |
| JuiYao Liu^87^ | 321 | English | Low | China | NA | Jan.21 to Apr.6 | 6 |

*Latitude include Low latitude(0°-30°)、Mid-latitude(30°-60°)、High latitude(60°-90°).

*Age is expressed as average, average±SD or median(IQR).

**Table S3.** Description of treatment of including studies.

| **Study** | **Therapies** | | | | **Outcome** | | |
| --- | --- | --- | --- | --- | --- | --- | --- |
|  | **Antiviral** | **Glucocorticoid** | **Oxygen Therapy** | **Mechanical Ventilation** | **Death** | **Discharged** | **Remained in Hospital** |
| **America** |  |  |  |  |  |  |  |
| CDC^1^ | - | - | - | - | 103700 | - | - |
| Shikha Garg^2^ | - | - | - | - | - | - | - |
| Jessica Ferguson^3^ | 32 | 5 | - | - | 6 | 62 | 4 |
| Safiya Richardson^4^ | - | - | 401 | 320 | 553 | 2081 | 3066 |
| Jeremy A W Gold^5^ | - | - | 126 | 103 | 48 | 233 | 24 |
| Youyou Duanmu^6^ | 11 | - | 6 | 6 | - | - | - |
| Laura C Myers^7^ | - | - | 118 | 118 | 50 | 321 | 6 |
| Parag Goyal^8^ | 17 | 46 | 393 | 393 | 40 | 260 | 93 |
| Geehan Suleyman^9^ | - | - | - | - | 72 | 108 | 283 |
| Molly Lin^10^ | - | - | - | - | - | - | - |
| Juan Pablo  Escalera-Antezana^11^ | - | - | - | - | - | - | - |
| Edgar Ortiz-Brizuela^12^ | 104 | - | - | - | - | - | - |
| **Europe** |  |  |  |  | - | - | - |
| Jerome R Lechien^13^ | - | - | - | - |  | 264 |  |
| Andrea Giacomelli^14^ | - | - | - | - | - | - | - |
| Corrado Lodigiani^15^ | - | - | - | - | - | - | - |
| Riccardo M Inciardi^16^ | - | - | 20 | 20 | 26 | - | - |
| Marta Colaneri^17^ | 31 | - | - | - | - | 14 | 30 |
| Fabio Ciceri^18^ | - | - | - | - | 95 | 291 | 24 |
| Jean-Christophe Lagier^19^ | - | - | - | - | - | - | - |
| Frédéric Lapostolle^20^ | - | - | - | - | - | - | - |
| Håkon Ihle-Hansen^21^ | - | - | - | - | - | 24 | - |
| Marlene M Speth^22^ | - | - | - | - | - | - | - |
| K Khalil^23^ | - | - | 34 | 34 | 58 | 162 |  |
| Annemarie B Docherty^24^ | - | - | - | - | 5165/  20133 | 8199/  20133 | 6769/  20133 |
| **Oceania** | - | - | - | - | - | - | - |
| 2019-nCoV National Incident Room Surveillance Team^25^ | - | - | - | - | 102 | - | - |
| 2019-nCoV National Incident Room Surveillance Team^26^ | - | - | - | - | 7 | - | - |
| **Asia** | - | - | - | - | - | - | - |
| Peong Gang Park^27^ | - | - | - | - | - | - | - |
| Eu Suk Kim^28^ | 19 | - | - | - | - | - | - |
| Kyung Soo Hong^29^ | 97 | 18 | 30 | 23 | 5 | 36 | 57 |
| Wonjun Ji^30^ | - | - | - | - | - | - | - |
| Yuzo Arima^31^ | - | - | - | - | - | - | - |
| Sakiko Tabata^32^ | - | - | - | - | - | - | - |
| Mohamad Nikpouraghdam^33^ | - | - | - | - | 239 | 2639 | 86 |
| Muayad A Merza^34^ | - | - | - | - | 0 | 15 | 0 |
| Barnaby Edward Young^35^ | - | - | 1 | 1 | - | - | - |
| Amit Aggarwal^36^ | - | - | - | - | - | - | - |
| WeiJie Guan^37^ | 394 | 220 | 81 | 81 | 15 | 55 | 1038 |
| China CDC^38^ | - | - | - | - | - | - | - |
| Adam Bernheim^39^ | - | - | - | - | - | - | - |
| Xiaolong Qi^40^ | - | - | - | - | - | - | - |
| Sijia Tian^41^ | - | - | - | - | 45 | 3 | 214 |
| Lijun Sun^42^ | 48 | 25 | 8 | 8 | - | 29 | 26 |
| Xiao Yu^43^ | - | - | - | - | - | - | - |
| Chunxia Cao^44^ | 54 | 21 | - | - | 3 | 132 | - |
| Yalei Shang^45^ | - | - | - | - | - | - | - |
| Qian Yu^46^ | - | - | - | - | 0 | 350 | - |
| Rui Huang^47^ | 196 | 64 | 9 | 9 | 0 | 37 | 165 |
| KaiCai Liu^48^ | 8 | - | - | - | - | 12 |  |
| Yan Ma^49^ | 47 | - | - | - | - | 45 | 2 |
| Wenjun Du^50^ | - | - | - | - | - | - | - |
| Liang Su^51^ | 23 | 0 | - | - | - | 23 | - |
| Xiaoming Li^52^ | - | - | - | - | - | - | - |
| K. Wang^53^ | - | - | - | - | - | - | - |
| Lei Pan^54^ | 184 | 80 | - | - | 37 | 168 | 0 |
| Kui Liu^55^ | 119 | 40 | 34 | 34 | 16 | 44 | 77 |
| Hong Zhang^56^ | 194 | - | 5 | 5 | 9 | 109 | 76 |
| Qiurong Ruan^57^ | 88 | 53 | 88 | 76 | 68 | 82 | 0 |
| Ling Mao^58^ | - | - | - | - | - | - | - |
| Gemin Zhang^59^ | - | - | - | - | - | - | - |
| Fei Zhou^60^ | 181 | 57 | 71 | 58 | 54 | 137 | 0 |
| Dawei Wang^61^ | 124 | 62 | 32 | 32 | - | - | - |
| Chen, Lei^62^ | - | - | - | - | 2 | 27 | - |
| Nanshan Chen^63^ | 75 | 19 | 29 | 17 | 11 | 31 | 57 |
| Chaolin Huang^64^ | 38 | 9 | 9 | 4 | 6 | 28 | 7 |
| JinJin Zhang^65^ | - | - | - | - | - | - | - |
| Fang Liu^66^ | 131 | - | - | - | - | - | - |
| Qiuxiang Yang^67^ | 136 | 108 | 20 | 20 | 23 | 99 | 14 |
| Chuan Qin^68^ | - | - | - | - | - | - | - |
| Guqin Zhang^69^ | - | - | - | - | - | - | - |
| Yun Feng^70^ | 319 | 127 | 77 | 73 | 38 | 403 | 35 |
| XinYing Zhao^71^ | 81 | 79 | 3 | 0 | 2 | 14 | 75 |
| Sheng Yin^72^ | - | - | - | - | - | - | - |
| Jiangshan Lian^73^ | 387 | 60 | 8 | 8 | - | - | - |
| Shufa Zheng^74^ | 96 | 78 | 15 | 10 | - | 96 | - |
| Qingqing Chen^75^ | 141 | 47 | 1 | 1 | - | 145 | - |
| Yu Shi^76^ | - | - | - | - | - | - | - |
| Wenjie Yang^77^ | 140 | 5 | 2 | 2 | 0 | 73 | 76 |
| Suxin Wan^78^ | 135 | 36 | 1 | 1 | 1 | 15 | 120 |
| Peng Chen^79^ | - | - | - | - | - | - | - |
| Yongli Zheng^80^ | - | - | - | - | - | - | - |
| Xi Xu^81^ | - | - | - | - | - | - | - |
| Ying Zhu^82^ | - | - | - | - | - | - | - |
| YingTao Zhang^83^ | - | - | - | - | 9 | 1329 | 12 |
| Yingxia Liu^84^ | 12 | 3 | 9 | 9 | - | - | - |
| Kai Liu^85^ | 53 | - | 17 | 10 | 3 | 53 | - |
| Iek Long Lo^86^ | 10 | 3 | - | - | - | 5 | 5 |
| JuiYao Liu^87^ | - | - | - | - | - | - | - |

**Table S4.** The relationship between the severity rate of COVID-19 patients with gender, age and geographical location

| **Variable** | | **Severity Rate** | **Male** | **Female** | **＜65 year** | **≥65 year** |
| --- | --- | --- | --- | --- | --- | --- |
| **Total** | | 24%  (20%-30%) | 26% (20%-32%) | 19% (14%-24%) | 16% (10%-23%) | 40%  (33%-47%) |
| **Longitude** | America | 20% (6%-41%) | 25% (3%-58%) | 16% (2%-39%) | 30% (23%-37%) | 54% (44%-63%) |
|  | Asia | 26% (23%-30%) | 27% (23%-32%) | 21% (17%-26%) | 14% (8%-20%) | 37%  (31%-44%) |
|  | Europe | 20% (12%-29%) | 19% (14%-24%) | 10% (5%-16%) | NR | NR |
|  | Oceania | 3%  (2%-3%) | 4%  (3%-4%) | 2%  (2%-3%) | NR | NR |
| **Latitude** | Low | 31% (21%-43%) | 33% (22%-44%) | 34% (17%-53%) | NR | NR |
|  | Middle | 25% (20%-31%) | 28% (22%-34%) | 19% (14%-24%) | 16%  (8%-25%) | 42% (35%-49%) |
|  | High | 21% (10%-36%) | NR | NR | NR | NR |

Note: NR represents no relevant data.

**Table S5.** Comorbidities and clinical manifestations of covid-19 patients in different regions.

|  | **Number of studies** | **Effect Size**  **(95%CL)** | **P** | **I^2^** | **Egger's** |
| --- | --- | --- | --- | --- | --- |
| **Comorbidities** | | | | | |
| **Hypertension** | 52 | 0.26(0.22-0.31) | ＜0.001 | 99.24% | 0.084 |
| **Continent** | 52 |  |  |  |  |
| America | 8 | 0.44(0.34-0.54) | ＜0.001 | 97.44% |  |
| Asia | 37 | 0.21(0.17-0.24) | ＜0.001 | 97.60% |  |
| Europe | 7 | 0.36(0.22-0.52) | ＜0.001 | 99.07% |  |
| **Latitude** | 48 |  |  |  |  |
| Low | 13 | 0.18(0.15-0.22) | ＜0.001 | 73.14% |  |
| Middle | 35 | 0.31(0.23-0.38) | ＜0.001 | 99.32% |  |
| **Diabetes** | 58 | 0.13(0.10-0.15) | ＜0.001 | 99.26% | 0.004 |
| **Continent** | 58 |  |  |  |  |
| America | 9 | 0.24(0.12-0.38) | ＜0.001 | 99.77% |  |
| Asia | 40 | 0.09(0.08-0.11) | ＜0.001 | 95.17% |  |
| Europe | 9 | 0.16(0.10-0.25) | ＜0.001 | 99.15% |  |
| **Latitude** | 52 |  |  |  |  |
| Low | 15 | 0.06(0.04-0.08) | ＜0.001 | 71.30% |  |
| Middle | 38 | 0.16(0.13-0.20) | ＜0.001 | 98.27% |  |
| High | 1 | 0.17(0.07-0.31) | NA | NA |  |
| **Cardiovascular** | 46 | 0.08(0.07-0.10) | ＜0.001 | 97.63% | 0.177 |
| **Continent** | 46 |  |  |  |  |
| America | 7 | 0.14(0.08-0.21) | ＜0.001 | 96.86% |  |
| Asia | 32 | 0.07(0.05-0.08) | ＜0.001 | 96.15% |  |
| Europe | 7 | 0.1(0.06-0.16) | ＜0.001 | 96.16% |  |
| **Latitude** | 42 |  |  |  |  |
| Low | 10 | 0.03(0.02-0.05) | ＜0.001 | 77.07% |  |
| Middle | 32 | 0.11(0.08-0.13) | ＜0.001 | 97.28% |  |
| **Lung disease** | 46 | 0.04(0.04-0.05) | ＜0.001 | 93.45% | 0.107 |
| **Continent** | 45 |  |  |  |  |
| America | 7 | 0.11(0.06-0.17) | ＜0.001 | 96.69% |  |
| Asia | 30 | 0.02(0.02-0.03) | ＜0.001 | 58.27% |  |
| Europe | 8 | 0.07(0.06-0.09) | 0.02 | 54.05% |  |
| **Latitude** | 39 |  |  |  |  |
| Low | 7 | 0.02(0.01-0.03) | 0.21 | 22.12% |  |
| Middle | 31 | 0.05(0.04-0.07) | ＜0.001 | 91.97% |  |
| High | 1 | 0.07(0.01-0.19) | NA | NA |  |
| **Cancer** | 48 | 0.03(0.02-0.04) | ＜0.001 | 91.63% | 0.974 |
| **Continent** | 46 |  |  |  |  |
| America | 6 | 0.05(0.04-0.07) | ＜0.001 | 73.87% |  |
| Asia | 32 | 0.02(0.01-0.03) | ＜0.001 | 88.81% |  |
| Europe | 8 | 0.07(0.04-0.11) | ＜0.001 | 92.94% |  |
| **Latitude** | 42 |  |  |  |  |
| Low | 9 | 0.01(0.01-0.02) | 0.31 | 16.16% |  |
| Middle | 32 | 0.04(0.03-0.05) | ＜0.001 | 91.65% |  |
| High | 1 | 0.14(0.05-0.29) | NA | NA |  |
| **Clinical manifestations** | | | | | |
| **Fever** | 75 | 0.71(0.67-0.75) | ＜0.001 | 99.45% | ＜0.001 |
| **Continent** | 75 |  |  |  |  |
| America | 11 | 0.62(0.53-0.70) | ＜0.001 | 99.10% |  |
| Asia | 53 | 0.76(0.70-0.81) | ＜0.001 | 96.47% |  |
| Europe | 10 | 0.59(0.37-0.80) | ＜0.001 | 99.82% |  |
| Oceania | 1 | 0.47(0.46-0.49) | NA | NA |  |
| **Latitude** | 67 |  |  |  |  |
| Low | 19 | 0.75(0.66-0.83) | ＜0.001 | 94.06% |  |
| Middle | 51 | 0.73(0.65-0.81) | ＜0.001 | 99.46% |  |
| High | 1 | 0.79(0.63-0.90) | NA | NA |  |
| **Fatigue** | 33 | 0.28(0.23-0.34) | ＜0.001 | 97.19% | 0.001 |
| **Continent** | 33 |  |  |  |  |
| America | 3 | 0.24(0.14-0.35) | NA | NA |  |
| Asia | 29 | 0.28(0.22-0.34) | ＜0.001 | 96.09% |  |
| Europe | 1 | 0.45(0.45-0.46) | NA | NA |  |
| **Latitude** | 32 |  |  |  |  |
| Low | 9 | 0.2(0.14-0.27) | ＜0.001 | 82.36% |  |
| Middle | 23 | 0.31(0.24-0.38) | ＜0.001 | 97.39% |  |
| **Myalgia** | 40 | 0.21(0.17-0.26) | ＜0.001 | 98.25% | 0.003 |
| **Continent** | 40 |  |  |  |  |
| America | 8 | 0.4(0.30-0.49) | ＜0.001 | 96.50% |  |
| Asia | 29 | 0.14(0.12-0.17) | ＜0.001 | 84.52% |  |
| Europe | 3 | 0.42(0.18-0.68) | NA | NA |  |
| **Latitude** | 36 |  |  |  |  |
| Low | 12 | 0.24(0.10-0.42) | ＜0.001 | 97.47% |  |
| Middle | 24 | 0.19(0.14-0.24) | ＜0.001 | 94.56% |  |
| **Cough** | 73 | 0.58(0.55-0.62) | ＜0.001 | 98.68% | 0.016 |
| **Continent** | 73 |  |  |  |  |
| America | 10 | 0.65(0.50-0.78) | ＜0.001 | 98.91% |  |
| Asia | 53 | 0.58(0.53-0.62) | ＜0.001 | 94.55% |  |
| Europe | 9 | 0.55(0.46-0.63) | ＜0.001 | 98.62% |  |
| Oceania | 1 | 0.69(0.68-0.70) | NA | NA |  |
| **Latitude** | 65 |  |  |  |  |
| Low | 18 | 0.61(0.53-0.69) | ＜0.001 | 91.97% |  |
| Middle | 46 | 0.57(0.52-0.62) | ＜0.001 | 97.79% |  |
| High | 1 | 0.67(0.50-0.80) | NA | NA |  |
| **Sputum production** | 29 | 0.24(0.20-0.27) | ＜0.001 | 94.50% | 0.721 |
| **Continent** | 29 |  |  |  |  |
| America | 1 | 0.21(0.12-0.32) | NA | NA |  |
| Asia | 24 | 0.24(0.19-0.29) | ＜0.001 | 93.70% |  |
| Europe | 4 | 0.24(0.16-0.34) | ＜0.001 | 97.80% |  |
| **Latitude** | 26 |  |  |  |  |
| Low | 6 | 0.23(0.14-0.34) | ＜0.001 | 90.88% |  |
| Middle | 20 | 0.25(0.20-0.29) | ＜0.001 | 93.21% |  |
| **Sore throat** | 42 | 0.18(0.15-0.22) | ＜0.001 | 98.89% | 0.76 |
| **Continent** | 42 |  |  |  |  |
| America | 7 | 0.26(0.16-0.38) | ＜0.001 | 96.18% |  |
| Asia | 29 | 0.16(0.12-0.20) | ＜0.001 | 91.02% |  |
| Europe | 5 | 0.16(0.02-0.40) | ＜0.001 | 99.69% |  |
| Oceania | 1 | 0.4(0.39-0.41) | NA | NA |  |
| **Latitude** | 37 |  |  |  |  |
| Low | 12 | 0.28(0.19-0.37) | ＜0.001 | 93.48% |  |
| Middle | 25 | 0.13(0.10-0.17) | ＜0.001 | 92.13% |  |
| **Dyspnea** | 54 | 0.26(0.20-0.32) | ＜0.001 | 99.67% | 0.62 |
| **Continent** | 54 |  |  |  |  |
| America | 8 | 0.48(0.33-0.64) | ＜0.001 | 98.92% |  |
| Asia | 37 | 0.17(0.11-0.23) | ＜0.001 | 97.55% |  |
| Europe | 9 | 0.46(0.29-0.64) | ＜0.001 | 99.70% |  |
| **Latitude** | 50 |  |  |  |  |
| Low | 12 | 0.15(0.07-0.25) | ＜0.001 | 94.86% |  |
| Middle | 37 | 0.28(0.18-0.38) | ＜0.001 | 99.75% |  |
| High | 1 | 0.69(0.53-0.82) | NA | NA |  |
| **Abdominal pain** | 19 | 0.06(0.04-0.07) | ＜0.001 | 95.43% | 0.704 |
| **Continent** | 19 |  |  |  |  |
| America | 4 | 0.09(0.06-0.12) | 0.01 | 72.13% |  |
| Asia | 12 | 0.03(0.01-0.04) | ＜0.001 | 61.68% |  |
| Europe | 3 | 0.13(0.06-0.21) | NA | NA |  |
| **Latitude** | 17 |  |  |  |  |
| Low | 7 | 0.06(0.01-0.13) | ＜0.001 | 88.32% |  |
| Middle | 10 | 0.05(0.02-0.08) | ＜0.001 | 90.92% |  |
| **Diarrhea** | 47 | 0.11(0.08-0.14) | ＜0.001 | 95.45% | 0.898 |
| **Continent** | 47 |  |  |  |  |
| America | 7 | 0.23(0.18-0.28) | ＜0.001 | 73.69% |  |
| Asia | 36 | 0.23(0.15-0.32) | ＜0.001 | 89.57% |  |
| Europe | 4 | 0.08(0.06-0.11) | ＜0.001 | 90.13% |  |
| **Latitude** | 43 |  |  |  |  |
| Low | 14 | 0.12(0.08-0.17) | ＜0.001 | 86.48% |  |
| Middle | 29 | 0.11(0.07-0.14) | ＜0.001 | 93.68% |  |
| **Nausea** | 27 | 0.1(0.08-0.13) | ＜0.001 | 94.82% | 0.826 |
| **Continent** | 26 |  |  |  |  |
| America | 6 | 0.18(0.13-0.25) | ＜0.001 | 93.53% |  |
| Asia | 17 | 0.06(0.04-0.09) | NA | NA |  |
| Europe | 3 | 0.19(0.17-0.21) | ＜0.001 | 87.78% |  |
| **Latitude** | 23 |  |  |  |  |
| Low | 9 | 0.08(0.03-0.15) | ＜0.001 | 90.74% |  |
| Middle | 14 | 0.11(0.07-0.16) | ＜0.001 | 92.85% |  |
| **Inappetence** | 17 | 0.24(0.18-0.31) | ＜0.001 | 97.24% | 0.008 |
| **Continent** | 17 |  |  |  |  |
| America | 2 | 0.15(0.13-0.18) | NA | NA |  |
| Asia | 11 | 0.23(0.14-0.33) | ＜0.001 | 95.42% |  |
| Europe | 4 | 0.35(0.28-0.42) | ＜0.001 | 94.68% |  |
| **Latitude** | 16 |  |  |  |  |
| Low | 3 | 0.11(0.03-0.23) | NA | NA |  |
| Middle | 13 | 0.27(0.20-0.33) | ＜0.001 | 95.25% |  |
| **Rhinorrhea** | 33 | 0.12(0.08-0.17) | ＜0.001 | 99.55% | 0.033 |
| **Continent** | 33 |  |  |  |  |
| America | 6 | 0.18(0.06-0.34) | ＜0.001 | 98.92% |  |
| Asia | 19 | 0.07(0.04-0.11) | ＜0.001 | 92.05% |  |
| Europe | 7 | 0.19(0.04-0.42) | ＜0.001 | 99.85% |  |
| Oceania | 1 | 0.25(0.24-0.26) | NA | NA |  |
| **Latitude** | 28 |  |  |  |  |
| Low | 8 | 0.14(0.05-0.27) | ＜0.001 | 95.00% |  |
| Middle | 20 | 0.1(0.04-0.17) | ＜0.001 | 99.18% |  |
| **Loss of smell/taste** | 12 | 0.24(0.14-0.35) | ＜0.001 | 99.68% | 0.16 |
| **Continent** | 12 |  |  |  |  |
| America | 4 | 0.04(0.01-0.09) | ＜0.001 | 90.64% |  |
| Asia | 4 | 0.11(0.08-0.15) | 0.27 | 24.18% |  |
| Europe | 3 | 0.83(0.60-0.97) | NA | NA |  |
| Oceania | 1 | 0.09(0.09-0.10) | NA | NA |  |
| **Latitude** | 9 |  |  |  |  |
| Low | 3 | 0.06(0.00-0.18) | NA | NA |  |
| Middle | 6 | 0.34(0.05-0.73) | ＜0.001 | 98.74% |  |
| **Asymptomatic** | 26 | 0.08(0.03-0.14) | ＜0.001 | 96.91% | 0.638 |
| **Continent** | 26 |  |  |  |  |
| Asia | 23 | 0.09(0.03-0.18) | ＜0.001 | 97.22% |  |
| Europe | 3 | 0.03(0.00-0.12) | NA | NA |  |
| **Latitude** | 24 |  |  |  |  |
| Low | 8 | 0.07(0.01-0.15) | ＜0.001 | 89.22% |  |
| Middle | 15 | 0.07(0.01-0.15) | ＜0.001 | 97.89% |  |
| High | 1 | 0.02(0.00-0.13) | NA | NA |  |

**Table S6** Compare the comorbidities of mild and severe COVID-19 patients.


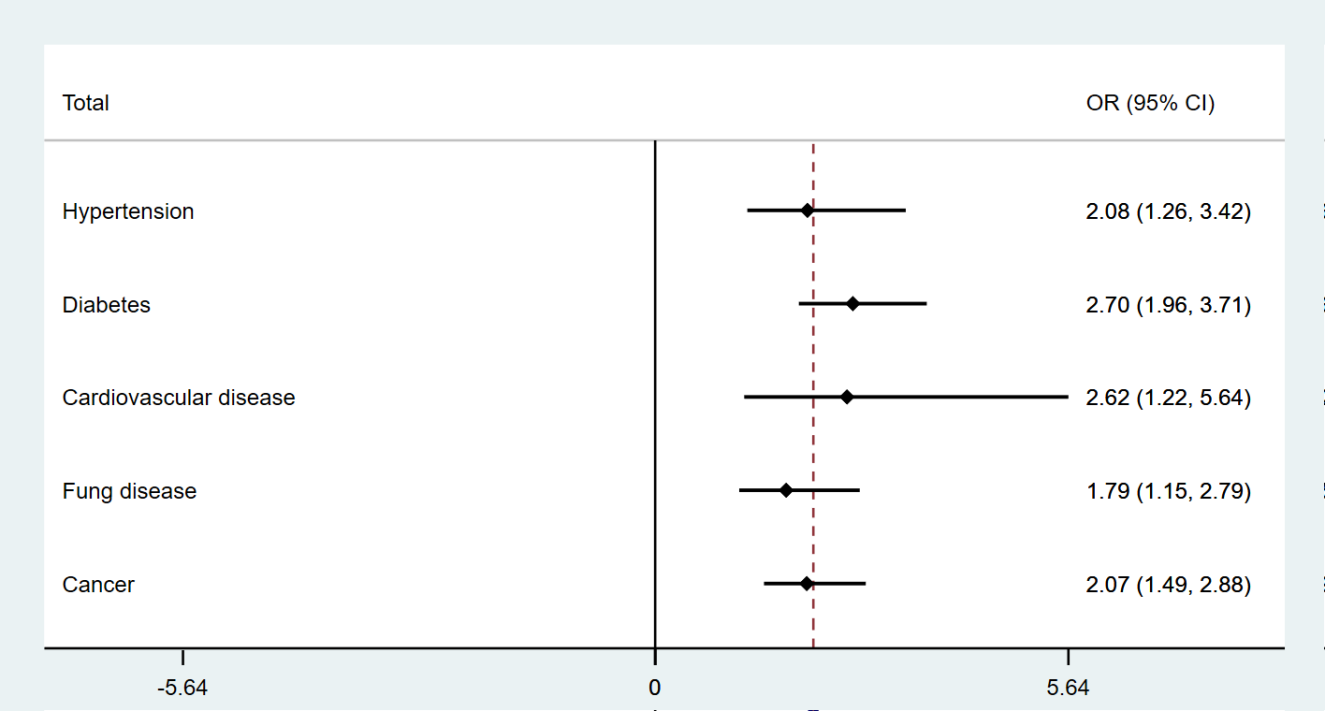


**Table S7.** Compare the comorbidities and Clinical manifestations of mild and severe COVID-19 patients in different regions.

|  | **Number of studies** | **OR** | **95%CL** | **P-value for heterogeneity** | **I^2^** | **P-value** | **Egger's** |
| --- | --- | --- | --- | --- | --- | --- | --- |
| **Comorbidities** | | | | | | | |
| **HBP** | 22 | 2.08 | 1.258-3.422 | ＜0.001 | 92.70% | 0.004 | 0.031 |
| **Continent** | 22 |  |  |  |  |  |  |
| America | 3 | 1.66 | 1.113-2.466 | 0.478 | 0.00% |  |  |
| Asia | 18 | 2.27 | 1.266-4.067 | ＜0.001 | 93.20% |  |  |
| Europe | 1 | 0.87 | 0.502-1.509 | NA | NA |  |  |
| **Latitude** | 22 |  |  |  |  |  |  |
| Low | 7 | 2.25 | 1.294-3.927 | ＜0.001 | 52.20% |  |  |
| Middle | 15 | 2.05 | 1.076-3.906 | 0.051 | 94.90% |  |  |
| **Diabetes** | 21 | 2.70 | 1.962-3.709 | ＜0.001 | 66.20% | ＜0.001 | 0.033 |
| **Continent** | 21 |  |  |  |  |  |  |
| America | 2 | 3.41 | 1.710-6.781 | 83.60% | 0.00% |  |  |
| Asia | 18 | 2.91 | 2.144-3.945 | 0.003 | 54.30% |  |  |
| Europe | 1 | 0.71 | 0.354-1.440 | NA | NA |  |  |
| **Latitude** | 21 |  |  |  |  |  |  |
| Low | 7 | 3.21 | 2.012-5.114 | 0.751 | 0.00% |  |  |
| Middle | 14 | 2.57 | 1.719-3.844 | ＜0.001 | 76.60% |  |  |
| **Cardiovascular** | 18 | 2.62 | 1.216-5.644 | ＜0.001 | 90.90% | 0.014 | 0.113 |
| **Continent** | 18 |  |  |  |  |  |  |
| America | 2 | 1.33 | 0.765-2.325 | 0.6 | 0.00% |  |  |
| Asia | 16 | 2.95 | 1.252-6.953 | ＜0.001 | 90.70% |  |  |
| **Latitude** | 18 |  |  |  |  |  |  |
| Low | 6 | 3.91 | 1.576-9.685 | 0.352 | 10.00% |  |  |
| Middle | 12 | 2.31 | 0.900-5.918 | ＜0.001 | 93.90% |  |  |
| **Lung Disease** | 14 | 1.79 | 1.148-2.794 | 0.287 | 15.20% | 0.01 | 0.303 |
| **Continent** | 14 |  |  |  |  |  |  |
| America | 2 | 1.45 | 0.813-2.575 | 0.637 | 0.00% |  |  |
| Asia | 12 | 2.11 | 1.146-3.883 | 0.202 | 24.60% |  |  |
| **Latitude** | 14 |  |  |  |  |  |  |
| Low | 3 | 2.85 | 0.141-57.642 | 0.051 | 66.30% |  |  |
| Middle | 11 | 1.67 | 1.141-2.446 | 0.511 | 0.00% |  |  |
| **Cancer** | 16 | 2.07 | 1.486-2.876 | 0.269 | 16.50% | ＜0.001 | 0.103 |
| **Continent** | 16 |  |  |  |  |  |  |
| America | 1 | 1.89 | 0.996-3.591 | NA | NA |  |  |
| Europe | 1 | 0.45 | 0.103-1.952 | NA | NA |  |  |
| Asia | 14 | 2.68 | 2.154-3.335 | 0.603 | 0.00% |  |  |
| **Latitude** | 16 |  |  |  |  |  |  |
| Low | 5 | 4.72 | 1.495-14.894 | 0.741 | 0.00% |  |  |
| Middle | 11 | 1.81 | 1.228-2.671 | 0.159 | 30.10% |  |  |
| **Clinical manifestations** | | | | | | | |
| **Fever** | 19 | 1.40 | 0.882-2.232 | ＜0.001 | 67.80% | 0.015 | 0.015 |
| **Continent** | 19 |  |  |  |  |  |  |
| America | 2 | 0.82 | 0.524-1.294 | 35.70% | 0.00% | 0.399 |  |
| Asia | 17 | 1.50 | 0.873-2.586 | ＜0.001 | 70.40% | 0.141 |  |
| **Latitude** | 19 |  |  |  |  |  |  |
| Low | 5 | 1.72 | 0.685-4.311 | 0.074 | 53.00% | 0.249 |  |
| Middle | 14 | 1.31 | 0.769-2.236 | ＜0.001 | 70.30% | 0.319 |  |
| **Fatigue** | 14 | 1.83 | 1.479-2.265 | 0.467 | 0.00% | ＜0.001 | 0.537 |
| **Latitude** | 14 |  |  |  |  |  |  |
| Low | 4 | 1.62 | 0.828-3.176 | 0.206 | 34.40% | 0.159 |  |
| Middle | 10 | 1.85 | 1.462-2.349 | 0.521 | 0.00% | ＜0.001 |  |
| **Myalgia** | 13 | 1.25 | 0.980-1.603 | 0.545 | 0.00% | 0.072 | 0.177 |
| **Continent** | 13 |  |  |  |  |  |  |
| America | 1 | 1.29 | 0.478-3.499 | NA | NA | 0.613 |  |
| Asia | 12 | 1.25 | 0.970-1.612 | 0.459 | 0.00% | 0.084 |  |
| **Latitude** | 13 |  |  |  |  |  |  |
| Low | 5 | 1.16 | 0.614-2.189 | 0.12 | 45.40% | 0.649 |  |
| Middle | 8 | 1.23 | 0.920-1.654 | 0.84 | 0.00% | 0.161 |  |
| **Cough** | 17 | 1.12 | 0.780-1.619 | 0 | 68.20% | 0.53 | 0.027 |
| **Continent** | 17 |  |  |  |  |  |  |
| America | 1 | 3.08 | 0.381-24.891 | NA | NA | 0.291 |  |
| Asia | 16 | 1.10 | 0.758-1.587 | ＜0.001 | 69.30% | 0.623 |  |
| **Latitude** | 17 |  |  |  |  |  |  |
| Low | 5 | 1.80 | 1.043-3.110 | 0.587 | 0.00% | 0.035 |  |
| Middle | 12 | 0.99 | 0.655-1.490 | ＜0.001 | 72.70% | 0.953 |  |
| **Sputum production** | 9 | 1.28 | 0.761-2.144 | 0.002 | 67.60% | 0.354 | 0.499 |
| **Latitude** | 9 |  |  |  |  |  |  |
| Low | 3 | 0.99 | 0.486-2.027 | 0.494 | 0.00% | 0.984 |  |
| Middle | 6 | 1.39 | 0.716-2.683 | 0 | 77.40% | 0.333 |  |
| **Sore throat** | 8 | 1.29 | 0.720-2.325 | 0.016 | 59.50% | 0.389 | 0.251 |
| **Continent** | 8 |  |  |  |  |  |  |
| America | 1 | 1.86 | 0.812-4.260 | NA | NA | 0.142 |  |
| Asia | 7 | 1.18 | 0.589-2.378 | 0.01 | 64.30% | 0.635 |  |
| **Latitude** | 8 |  |  |  |  |  |  |
| Low | 2 | 0.31 | 0.003-29.035 | 0.002 | 89.60% | 0.611 |  |
| Middle | 6 | 1.41 | 0.820-2.432 | 0.107 | 44.80% | 0.214 |  |
| **Dyspnea** | 12 | 6.49 | 3.596-11.715 | ＜0.001 | 78.00% | ＜0.001 | 0.016 |
| **Continent** | 12 |  |  |  |  |  |  |
| America | 2 | 2.00 | 1.291-3.082 | 86.50% | 0.00% | 0.002 |  |
| Asia | 10 | 9.55 | 4.670-19.543 | ＜0.001 | 77.10% | ＜0.001 |  |
| **Latitude** | 12 |  |  |  |  |  |  |
| Low | 4 | 2.46 | 1.037-5.857 | 0.003 | 78.70% | 0.018 |  |
| Middle | 8 | 5.68 | 3.045-10.605 | ＜0.001 | 81.10% | ＜0.001 |  |
| **Abdominal pain** | 9 | 2.22 | 1.167-4.229 | 0.242 | 22.60% | 0.015 | 0.765 |
| **Continent** | 9 |  |  |  |  |  |  |
| America | 1 | 3.61 | 1.213-10.724 | NA | NA | 0.021 |  |
| Asia | 8 | 1.95 | 0.907-4.184 | 0.217 | 26.60% | 0.087 |  |
| **Latitude** | 9 |  |  |  |  |  |  |
| Low | 3 | 0.96 | 0.163-5.580 | 0.037 | 69.80% | 0.959 |  |
| Middle | 6 | 2.88 | 1.450-5.727 | 0.726 | 0.00% | 0.003 |  |
| **Diarrhea** | 15 | 1.19 | 0.794-1.781 | 0.005 | 55.30% | 0.4 | 0.82 |
| **Continent** | 15 |  |  |  |  |  |  |
| America | 13 | 0.94 | 0.614-1.444 | 48.60% | 0.00% | 0.784 |  |
| Asia | 2 | 1.24 | 0.739-2.079 | 0.003 | 60.10% | 0.416 |  |
| **Latitude** | 15 |  |  |  |  |  |  |
| Low | 5 | 2.46 | 1.037-5.857 | 0.055 | 56.90% | 0.041 |  |
| Middle | 10 | 0.90 | 0.643-1.270 | 0.205 | 26.00% | 0.56 |  |
| **Nausea** | 5 | 1.67 | 0.863-3.223 | 0.208 | 32.00% | 0.128 | 0.879 |
| **Latitude** | 5 |  |  |  |  |  |  |
| Low | 2 | 1.13 | 0.276-4.598 | 15.30% | 51.00% | 0.869 |  |
| Middle | 3 | 2.08 | 0.761-5.691 | 17.30% | 43.10% | 0.153 |  |
| **Inappetence** | 11 | 2.41 | 1.344-4.326 | ＜0.001 | 80.50% | 0.003 | 0.07 |
| **Latitude** | 11 |  |  |  |  |  |  |
| Low | 2 | 6.86 | 0.592-79.536 | 0.086 | 66.00% | 0.124 |  |
| Middle | 9 | 2.16 | 1.138-4.089 | ＜0.001 | 82.30% | 0.018 |  |
| **Rhinorrhea** | 5 | 0.84 | 0.465-1.525 | 0.643 | 0.00% | 0.57 | 0.344 |
| **Continent** | 5 |  |  |  |  |  |  |
| America | 1 | 0.50 | 0.188-1.336 | NA | NA | 0.167 |  |
| Asia | 4 | 1.14 | 0.539-2.402 | 0.847 | 0.00% | 0.735 |  |
| **Latitude** | 5 |  |  |  |  |  |  |
| Low | 3 | 0.75 | 0.389-1.432 | 45.50% | 0.00% | 0.379 |  |
| Middle | 2 | 1.52 | 0.359-6.455 | 69.50% | 0.00% | 0.568 |  |

**Included studies：**

1. Stokes, Erin K et al., “Coronavirus Disease 2019 Case Surveillance - United States, January 22-May 30, 2020,” MMWR. Morbidity and mortality weekly report, vol. 69, no. 24, pp. 759-765, 2020.

2. Garg, Shikha et al., “Hospitalization Rates and Characteristics of Patients Hospitalized with Laboratory-Confirmed Coronavirus Disease 2019 - COVID-NET, 14 States, March 1-30, 2020,” MMWR. Morbidity and mortality weekly report, vol. 69, no. 15, pp. 458-464, 2020.

3. Ferguson, Jessica et al., “Characteristics and Outcomes of Coronavirus Disease Patients under Nonsurge Conditions, Northern California, USA, March-April 2020,” Emerging infectious diseases, vol. 26, no. 8, pp 1679-1685, 2020.

4. Richardson, Safiya et al., “Presenting Characteristics, Comorbidities, and Outcomes Among 5700 Patients Hospitalized With COVID-19 in the New York City Area,” JAMA, vol. 323, no. 20, pp. 2052-2059, 2020.

5. Gold, Jeremy A W et al., “Characteristics and Clinical Outcomes of Adult Patients Hospitalized with COVID-19 - Georgia, March 2020,” MMWR. Morbidity and mortality weekly report, vol. 69, no. 18, pp. 545-550, 2020.

6. Duanmu, Youyou et al., “Characteristics of Emergency Department Patients With COVID-19 at a Single Site in Northern California: Clinical Observations and Public Health Implications,” Academic emergency medicine, vol. 27, no. 6, pp. 505-509, 2020.

7. Myers, Laura C et al., “Characteristics of Hospitalized Adults With COVID-19 in an Integrated Health Care System in California,” JAMA, vol. 323, no. 21, pp. 2195-2198, 2020.

8. Goyal, Parag et al., “Clinical Characteristics of Covid-19 in New York City,” The New England journal of medicine, vol. 382, no. 24, pp. 2372-2374, 2020.

9. Suleyman, Geehan et al., “Clinical Characteristics and Morbidity Associated With Coronavirus Disease 2019 in a Series of Patients in Metropolitan Detroit,” JAMA, vol. 3, no. 6, Article ID e2012270, 2020.

10. Lin, Molly et al., “What can early Canadian experience screening for COVID-19 teach us about how to prepare for a pandemic?,” CMAJ, vol. 192, no. 12, pp. E314-E318, 2020.

11. Escalera-Antezana, Juan Pablo et al., “Clinical features of the first cases and a cluster of Coronavirus Disease 2019 (COVID-19) in Bolivia imported from Italy and Spain,” Travel medicine and infectious disease, vol. 35, Article ID 101653, 2020.

12. Ortiz-Brizuela, Edgar et al., “CLINICAL AND EPIDEMIOLOGICAL CHARACTERISTICS OF PATIENTS DIAGNOSED WITH COVID-19 IN A TERTIARY CARE CENTER IN MEXICO CITY: A PROSPECTIVE COHORT STUDY,” Revista de investigacion clinica; organo del Hospital de Enfermedades de la Nutricion, vol. 72, no. 3, pp. 165-177, 2020.

13. Lechien, Jerome R et al., “Clinical and epidemiological characteristics of 1420 European patients with mild-to-moderate coronavirus disease 2019,” Journal of internal medicine, vol. 288,no. 3, pp. 335-344, 2020.

14. Giacomelli, Andrea et al., “Self-reported Olfactory and Taste Disorders in Patients With Severe Acute Respiratory Coronavirus 2 Infection: A Cross-sectional Study,” Clinical infectious diseases, vol. 71, no. 15, pp. 889-890, 2020.

15. Lodigiani, Corrado et al., “Venous and arterial thromboembolic complications in COVID-19 patients admitted to an academic hospital in Milan, Italy,” Thrombosis research, vol. 191, pp. 9-14, 2020.

16. Inciardi, Riccardo M et al., “Characteristics and outcomes of patients hospitalized for COVID-19 and cardiac disease in Northern Italy,” European heart journal, vol. 41, no. 19 , pp. 1821-1829, 2020.

17. Colaneri, Marta et al., “Clinical characteristics of coronavirus disease (COVID-19) early findings from a teaching hospital in Pavia, North Italy, 21 to 28 February 2020,” Euro surveillance, vol. 25, no. 16, Article ID 101791, 2020.

18. Ciceri, Fabio et al., “Early predictors of clinical outcomes of COVID-19 outbreak in Milan, Italy,” Clinical immunology (Orlando, Fla.), vol. 217, Article ID 101791, 2020.

19. Lagier, Jean-Christophe et al., “Outcomes of 3,737 COVID-19 patients treated with hydroxychloroquine/azithromycin and other regimens in Marseille, France: A retrospective analysis,” Travel medicine and infectious disease, vol. 36, Article ID 101791, 2020.

20. Lapostolle, Frédéric et al., “Clinical features of 1487 COVID-19 patients with outpatient management in the Greater Paris: the COVID-call study,” Internal and emergency medicine, vol. 15, no. 5, pp. 813-817, 2020.

21. Ihle-Hansen, Håkon et al., “COVID-19: Symptoms, course of illness and use of clinical scoring systems for the first 42 patients admitted to a Norwegian local hospital,” Tidsskrift for den Norske laegeforening, vol. 140, no. 7, Article ID 10.4045, 2020.

22. Speth, Marlene M et al., “Olfactory Dysfunction and Sinonasal Symptomatology in COVID-19: Prevalence, Severity, Timing, and Associated Characteristics,” Otolaryngology head and neck surgery, vol. 163, no. 1, pp. 114-120, 2020.

23. Khalil, K et al., “Clinical characteristics and 28-day mortality of medical patients admitted with COVID-19 to a central London teaching hospital,” The Journal of infection, vol. 81, no. 3, pp. e85-e89, 2020.

24. Docherty, Annemarie B et al., “Features of 20 133 UK patients in hospital with covid-19 using the ISARIC WHO Clinical Characterisation Protocol: prospective observational cohort study,” BMJ (Clinical research ed.), vol. 369, Article ID 1985, 2020.

25. COVID-19 National Incident Room Surveillance Team, “COVID-19, Australia: Epidemiology Report 17 (Fortnightly reporting period ending 24 May 2020),” Communicable diseases intelligence (2018), vol. 44, no. 10, Article ID 33321, 2020.

26. COVID-19 National Incident Room Surveillance Team, “COVID-19, Australia: Epidemiology Report 16 (Reporting week to 23:59 AEST 17 May 2020),” Communicable diseases intelligence (2018), vol. 44, no. 10, Article ID 33321, 2020.

27. Park, Peong Gang et al., “Out-of-Hospital Cohort Treatment of Coronavirus Disease 2019 Patients with Mild Symptoms in Korea: an Experience from a Single Community Treatment Center,” Journal of Korean medical science, vol. 35, no. 13, Article ID 140, 2020.

28. Kim, Eu Suk et al., “Clinical Course and Outcomes of Patients with Severe Acute Respiratory Syndrome Coronavirus 2 Infection: a Preliminary Report of the First 28 Patients from the Korean Cohort Study on COVID-19,” Journal of Korean medical science, vol. 35, no. 13, Article ID 142, 2020.

29. Hong, Kyung Soo et al., “Clinical Features and Outcomes of 98 Patients Hospitalized with SARS-CoV-2 Infection in Daegu, South Korea: A Brief Descriptive Study,” Yonsei medical journal, vol. 61, no. 5, pp. 431-437, 2020.

30. Ji, Wonjun et al., “Effect of Underlying Comorbidities on the Infection and Severity of COVID-19 in Korea: a Nationwide Case-Control Study,” Journal of Korean medical science, vol. 35, no. 25, Article ID 237, 2020.

31. Arima, Yuzo et al., “Severe Acute Respiratory Syndrome Coronavirus 2 Infection among Returnees to Japan from Wuhan, China, 2020,” Emerging infectious diseases, vol. 26, no. 7, pp. 1596–1600, 2020.

32. Tabata, Sakiko et al., “Clinical characteristics of COVID-19 in 104 people with SARS-CoV-2 infection on the Diamond Princess cruise ship: a retrospective analysis,” The Lancet. Infectious diseases, vol. 20, no. 9, pp. 1043-1050, 2020.

33. Nikpouraghdam, Mohamad et al., “Epidemiological characteristics of coronavirus disease 2019 (COVID-19) patients in IRAN: A single center study,” Journal of clinical virology, vol. 127, Article ID 104378, 2020.

34. Merza, Muayad A et al., “COVID-19 outbreak in Iraqi Kurdistan: The first report characterizing epidemiological, clinical, laboratory, and radiological findings of the disease,” Diabetes & metabolic syndrome, vol. 14, no. 4,pp. 547-554, 2020.

35. Young, Barnaby Edward et al., “Epidemiologic Features and Clinical Course of Patients Infected With SARS-CoV-2 in Singapore,” JAMA, vol. 323, no. 15, pp. 1488-1494, 2020.

36. Aggarwal, Amit et al., “Clinical and Epidemiological Features of SARS-CoV-2 Patients in SARI Ward of a Tertiary Care Centre in New Delhi,” The Journal of the Association of Physicians of India, vol. 68, no. 7, pp. 19-26, 2020.

37. Eastin C, Eastin TJJoEM et al., “Clinical Characteristics of Coronavirus Disease 2019 in China”. vol. 58, no. 4, pp. 711-712, 2020.

38. Epidemiology Working Group for NCIP Epidemic Response, “The epidemiological characteristics of an outbreak of 2019 novel coronavirus diseases (COVID-19) in China,” Chinese Center for Disease Control and Prevention, vol. 41, no. 2, pp. 145-151, 2020.

39. Bernheim, Adam et al., “Chest CT Findings in Coronavirus Disease-19 (COVID-19): Relationship to Duration of Infection,” Radiology, vol. 295, no. 3, Article ID 200463, 2020.

40. Qi, Xiaolong et al., “Multicenter analysis of clinical characteristics and outcomes in patients with COVID-19 who develop liver injury,” Journal of hepatology, vol. 73, no. 2, pp. 455-458, 2020.

41. Tian, Sijia et al., “Characteristics of COVID-19 infection in Beijing,” The Journal of infection, vol. 80, no. 4, pp. 401-406, 2020.

42. Sun, Lijun et al., “Clinical features of patients with coronavirus disease 2019 from a designated hospital in Beijing, China,” Journal of medical virology, vol. 92,no. 10, pp. 2055-2066, 2020.

43. Yu, Xiao et al., “Epidemiological and clinical characteristics of 333 confirmed cases with coronavirus disease 2019 in Shanghai, China,” Transboundary and emerging diseases, vol. 67, no. 4, pp. 1697-1707, 2020.

44. Cao, Chunxia et al., “Epidemiologic Features of 135 Patients With Coronavirus Disease (COVID-19) in Tianjin, China,” Disaster medicine and public health preparedness, vol. 14, no. 5, pp. 630-634, 2020.

45. Shang, Yalei et al., “Clinical characteristics and changes of chest CT features in 307 patients with common COVID-19 pneumonia infected SARS-CoV-2: A multicenter study in Jiangsu, China,” International journal of infectious diseases, vol. 96, pp. 157-162, 2020.

46. Yu, Qian et al., “Multicenter cohort study demonstrates more consolidation in upper lungs on initial CT increases the risk of adverse clinical outcome in COVID-19 patients,” Theranostics, vol. 10, no. 12, pp. 5641-5648, 2020.

47. Huang, Rui et al., “Clinical findings of patients with coronavirus disease 2019 in Jiangsu province, China: A retrospective, multi-center study,” PLoS neglected tropical diseases, vol. 14, no. 5, Article ID 0008280, 2020.

48. Liu, Kai-Cai et al., “CT manifestations of coronavirus disease-2019: A retrospective analysis of 73 cases by disease severity,” European journal of radiology, vol. 126, Article ID 108941, 2020.

49. Ma, Yan et al., “Characteristics of asymptomatic patients with SARS-CoV-2 infection in Jinan, China,” Microbes and infection, vol. 22, no. 4, pp. 212-217, 2020.

50. Du, Wenjun et al., “Clinical characteristics of COVID-19 in children compared with adults in Shandong Province, China,” Infection, vol. 48, no. 3, pp. 445-452, 2020.

51. Su, Liang et al., “The different clinical characteristics of corona virus disease cases between children and their families in China - the character of children with COVID-19,” Emerging microbes & infections, vol. 9, no. 1, pp. 707-713, 2020.

52. Li, Xiaoming et al., “CT imaging changes of corona virus disease 2019(COVID-19): a multi-center study in Southwest China,” Journal of translational medicine, vol. 18, no. 1, pp.154, 2020.

53. Wang, K et al., “Imaging manifestations and diagnostic value of chest CT of coronavirus disease 2019 (COVID-19) in the Xiaogan area,” Clinical radiology, vol. 75, no. 5, pp. 341-347, 2020.

54. Pan, Lei et al., “Clinical Characteristics of COVID-19 Patients With Digestive Symptoms in Hubei, China: A Descriptive, Cross-Sectional, Multicenter Study,” The American journal of gastroenterology, vol. 115, no. 5, pp. 766-773, 2020.

55. Liu, Kui et al., “Clinical characteristics of novel coronavirus cases in tertiary hospitals in Hubei Province,” Chinese medical journal, vol. 133, no. 9, pp. 1025-1031, 2020.

56. Zhang, Hong et al., “Clinical characteristics of 194 cases of COVID-19 in Huanggang and Taian, China,” Infection, vol. 48, no. 5, pp, 687-694, 2020.

57. Ruan, Qiurong et al., “Clinical predictors of mortality due to COVID-19 based on an analysis of data of 150 patients from Wuhan, China,” Intensive care medicine, vol. 46, no. 5, pp. 846-848, 2020.

58. Mao, Ling et al., “Neurologic Manifestations of Hospitalized Patients With Coronavirus Disease 2019 in Wuhan, China,” JAMA neurology, vol. 77, no. 6, pp. 683-690, 2020.

59. Zhang, Gemin et al., “Analysis of clinical characteristics and laboratory findings of 95 cases of 2019 novel coronavirus pneumonia in Wuhan, China: a retrospective analysis,” Respiratory research, vol. 21, no. 1, pp. 74, 2020.

60. Zhou, Fei et al., “Clinical course and risk factors for mortality of adult inpatients with COVID-19 in Wuhan, China: a retrospective cohort study,” Lancet (London, England), vol. 395, no. 10229, pp. 1054-1062, 2020.

61. Wang, Dawei et al., “Clinical Characteristics of 138 Hospitalized Patients With 2019 Novel Coronavirus-Infected Pneumonia in Wuhan, China,” JAMA, vol. 323, no. 11, pp. 1061-1069, 2020.

62. Chen, L et al., “Analysis of clinical features of 29 patients with 2019 novel coronavirus pneumonia,” Chinese journal of tuberculosis and respiratory diseases, vol. 43, no. 3, pp. 203-208, 2020.

63. Chen, Nanshan et al., “Epidemiological and clinical characteristics of 99 cases of 2019 novel coronavirus pneumonia in Wuhan, China: a descriptive study,” Lancet (London, England), vol. 395, no. 10223, pp. 507-513, 2020.

64. Huang, Chaolin et al., “Clinical features of patients infected with 2019 novel coronavirus in Wuhan, China,” Lancet (London, England), vol. 395,no. 10223, pp. 497-506. 2020.

65. Zhang, Jin-Jin et al., “Clinical characteristics of 140 patients infected with SARS-CoV-2 in Wuhan, China,” Allergy, vol. 75, no. 7, pp. 1730-1741, 2020.

66. Liu, Fang et al., “Prognostic value of interleukin-6, C-reactive protein, and procalcitonin in patients with COVID-19,” Journal of clinical virology : the official publication of the Pan American Society for Clinical Virology, vol. 127, Article ID 104370, 2020.

67. Yang, Qiuxiang et al., “Analysis of the clinical characteristics, drug treatments and prognoses of 136 patients with coronavirus disease 2019,” Journal of clinical pharmacy and therapeutics, vol. 45, no. 4, pp. 609-616, 2020.

68. Qin, Chuan et al., “Dysregulation of Immune Response in Patients With Coronavirus 2019 (COVID-19) in Wuhan, China,” Clinical infectious diseases : an official publication of the Infectious Diseases Society of America, vol. 71, no. 15, pp. 762-768, 2020.

69. Zhang, Guqin et al., “Clinical features and short-term outcomes of 221 patients with COVID-19 in Wuhan, China,” Journal of clinical virology : the official publication of the Pan American Society for Clinical Virology, vol. 127, Article ID 104364, 2020.

70. Feng, Yun et al., “COVID-19 with Different Severities: A Multicenter Study of Clinical Features,” American journal of respiratory and critical care medicine, vol. 201, no. 11, pp. 1380-1388, 2020.

71. Zhao, Xin-Ying et al. “Clinical characteristics of patients with 2019 coronavirus disease in a non-Wuhan area of Hubei Province, China: a retrospective study.” BMC infectious diseases vol. 20,1 311. 29 Apr. 2020.

72. Yin, Sheng et al., “The implications of preliminary screening and diagnosis: Clinical characteristics of 33 mild patients with SARS-CoV-2 infection in Hunan, China,” Journal of clinical virology : the official publication of the Pan American Society for Clinical Virology, vol. 128, Article ID 104397, 2020.

73. Lian, Jiangshan et al., “Epidemiological, clinical, and virological characteristics of 465 hospitalized cases of coronavirus disease 2019 (COVID-19) from Zhejiang province in China,” Influenza and other respiratory viruses, vol. 14, no. 5, pp. 564-574, 2020.

74. Zheng, Shufa et al., “Viral load dynamics and disease severity in patients infected with SARS-CoV-2 in Zhejiang province, China, January-March 2020: retrospective cohort study,” BMJ (Clinical research ed.), vol. 369, Article ID 1443, 2020.

75. Chen, Qingqing et al., “Clinical characteristics of 145 patients with corona virus disease 2019 (COVID-19) in Taizhou, Zhejiang, China,” Infection, vol. 48, no. 4, pp. 543-551, 2020.

76. Shi, Yu et al., “Host susceptibility to severe COVID-19 and establishment of a host risk score: findings of 487 cases outside Wuhan,” Critical care (London, England), vol. 24, no. 1, pp. 108, 2020.

77. Yang, Wenjie et al., “Clinical characteristics and imaging manifestations of the 2019 novel coronavirus disease (COVID-19):A multi-center study in Wenzhou city, Zhejiang, China,” The Journal of infection, vol. 80, no. 4, pp. 388-393, 2020.

78. Wan, Suxin et al., “Clinical features and treatment of COVID-19 patients in northeast Chongqing,” Journal of medical virology, vol. 92, no. 7, pp. 797-806, 2020.

79. Chen, Peng et al., “Epidemiological and clinical characteristics of 136 cases of COVID-19 in main district of Chongqing,” Journal of the Formosan Medical Association, vol. 119, no. 7, pp. 1180-1184, 2020.

80. Zheng, Yongli et al., “Epidemiological characteristics and clinical features of 32 critical and 67 noncritical cases of COVID-19 in Chengdu,” Journal of clinical virology : the official publication of the Pan American Society for Clinical Virology, vol. 127, Article ID 104366, 2020.

81.Xu, Xi et al. “Imaging and clinical features of patients with 2019 novel coronavirus SARS-CoV-2,” European journal of nuclear medicine and molecular imaging, vol. 47,no 5, pp. 1275-1280, 2020.

82. Zhu, Ying et al., “Clinical and CT imaging features of 2019 novel coronavirus disease (COVID-19),” The Journal of infection. vol. 81,no. 1,pp. 147-178, 2020.

83. Zhang, YT et al., “Clinical outcomes of COVID-19 cases and influencing factors in Guangdong province,” Zhonghua liu xing bing xue. vol. 41,no. 12, pp. 1999-2004, 2020.

84. Liu, Yingxia et al., “Clinical and biochemical indexes from 2019-nCoV infected patients linked to viral loads and lung injury,” Science China. Life sciences, vol. 63, no. 3, pp. 364-374, 2020.

85. Liu, Kai et al, “Clinical features of COVID-19 in elderly patients: A comparison with young and middle-aged patients,” The Journal of infection, vol. 80, no. 6, pp. e14-e18, 2020.

86. Lo, Iek Long et al., “Evaluation of SARS-CoV-2 RNA shedding in clinical specimens and clinical characteristics of 10 patients with COVID-19 in Macau,” International journal of biological sciences, vol. 16, no. 10, pp. 1698-1707, 2020.

87. Liu, Jui-Yao et al., “Analysis of Imported Cases of COVID-19 in Taiwan: A Nationwide Study,” International journal of environmental research and public health, vol. 17, no. 9, pp. 3311, 2020.
